# Supplementary material for: A 3D adrenocortical carcinoma tumor platform for preclinical modeling of drug response and matrix metalloproteinase activity
Source: Sci Rep. 2023 Sep 19;13:15508. doi: 10.1038/s41598-023-42659-0 (PMC10509170; doi:10.1038/s41598-023-42659-0)
Supplement: Supplementary file 1 — Supplementary Figures. [file 41598_2023_42659_MOESM1_ESM.docx]

**Supplementary Information for:**

A 3D adrenocortical carcinoma tumor platform for preclinical modeling of drug response and matrix metalloproteinase activity

Priya H. Dedhia^1,2,3,^*, Hemamylammal Sivakumar^4^, Marco A. Rodriguez^4^, Kylie G. Nairon^4^, Joshua M. Zent^4^, Xuguang Zheng^1^, Katie Jones^4^, Liudmila Popova^1^, Jennifer L. Leight^3,4,5,^*, and Aleksander Skardal^3,4,5,^*

^1^Division of Surgical Oncology, The Ohio State University and Arthur G. James Comprehensive Cancer Center, Columbus, Ohio, USA.

^2^Translational Therapeutics Program, The Ohio State University and Arthur G. James Comprehensive Cancer Center, Columbus, Ohio, USA.

^3^Center for Cancer Engineering, The Ohio State University, Columbus, Ohio, USA.

^4^Department of Biomedical Engineering, College of Engineering, The Ohio State University, Columbus, Ohio, USA.

^5^Cancer Biology Program, The Ohio State University and Arthur G. James Comprehensive Cancer Center, Columbus, Ohio, USA.

*Correspondence

Priya Dedhia, MD, PhD

Division of Surgical Oncology

The Ohio State University and Arthur G. James Comprehensive Cancer Center

816 Biomedical Research Tower

460 W. 12^th^ Ave

Columbus, OH 43210

priya.dedhia@osumc.edu

Jennifer Leight, PhD

Department of Biomedical Engineering

The Ohio State University

886 Biomedical Research Tower

460 W. 12^th^ Ave

Columbus, OH 43210

leight.1@osu.edu

Aleksander Skardal, PhD

Department of Biomedical Engineering

The Ohio State University

3022 Fontana Labs

140 W. 19^th^ Ave

Columbus, OH 43210

Tel: 614-247-6643

skardal.1@osu.edu

**Supplementary Figures**

**
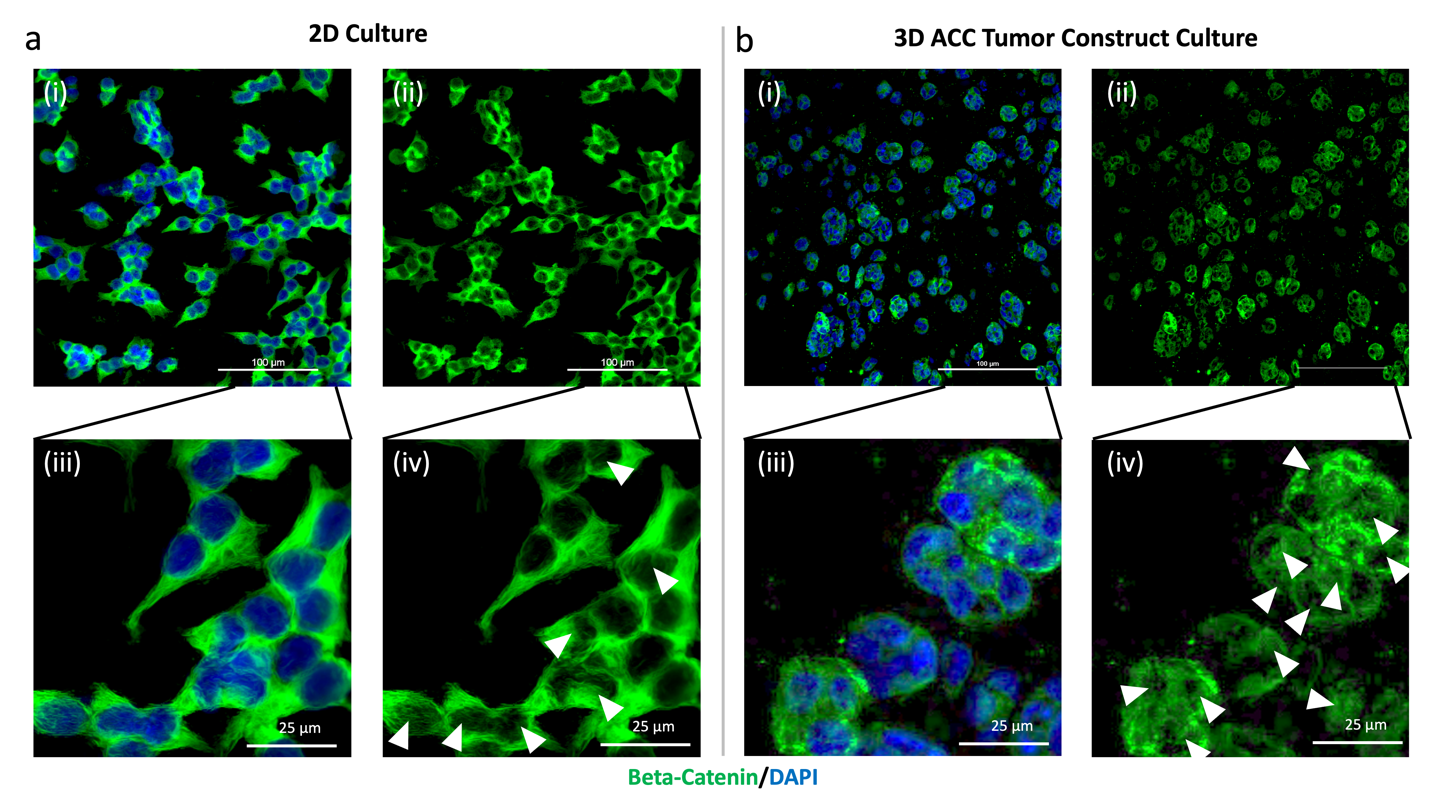
**

**Supplementary Figure 1. β-catenin expression is found in the cytoplasm and nuclei indicating activation of the Wnt pathway in 2D and 3D cultures.** Fluorescent imaging of NCI-H295R ACC cells cultured a) on 2D tissue culture plastic or b) in 3D tumor construct cultures. For both a) and b) panels, (i) shows the β-catenin expression (green) with stained nuclei (DAPI-blue) and (ii) β-catenin expression only (green). Analogous higher resolution images (iii) show β-catenin expression (green) with stained nuclei (DAPI - blue) and (iv) β-catenin expression only (green). Both cytoplasmic and nuclear β-catenin is observed in both 3D and 2D conditions. Arrows – positive nuclear β-catenin. Scale bars: 100 µm for (i) and (ii) panels; 25 µm for (iii) and (iv) panels.


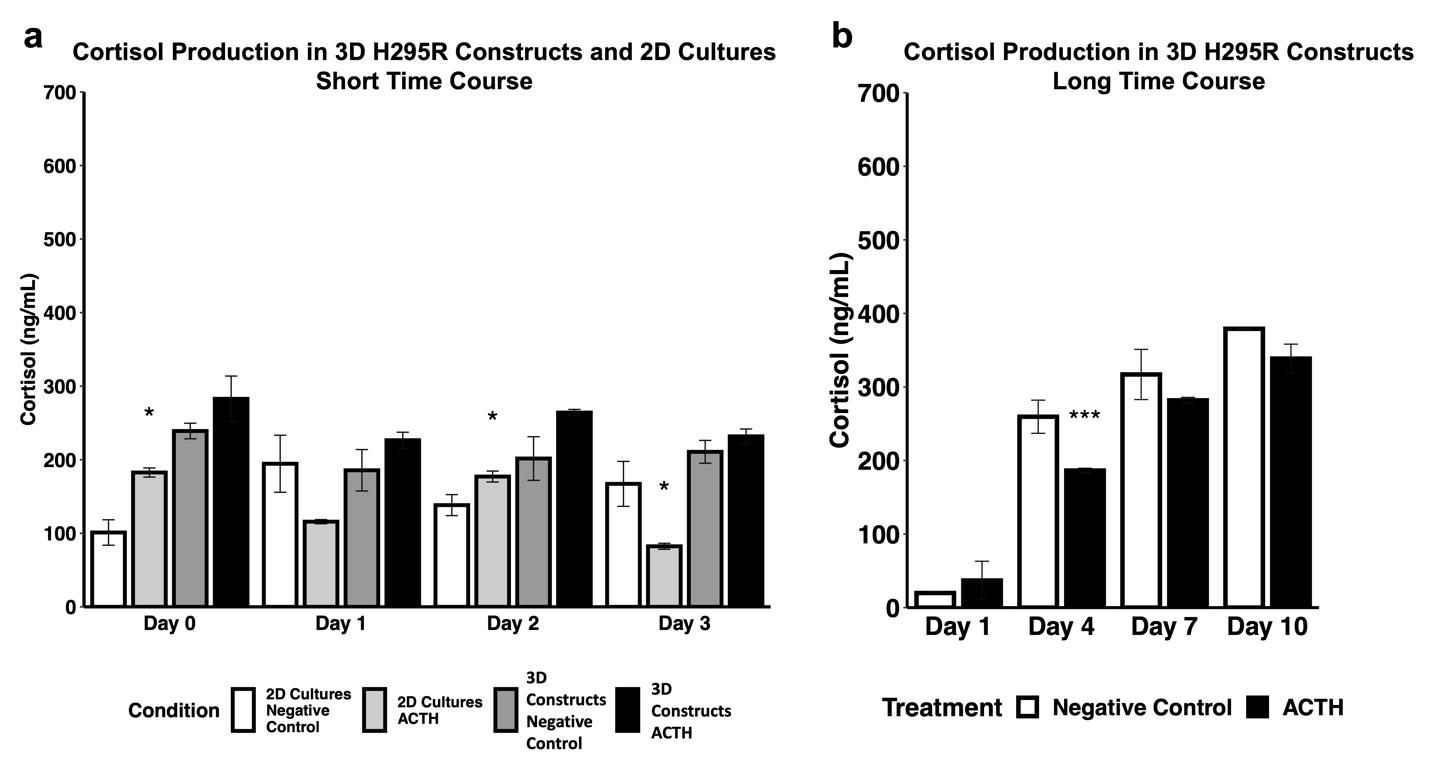


**Supplementary Figure 2. 3D ACC tumor constructs do not increase cortisol secretion in response to ACTH.** 3D ACC tumor constructs and 2D cell cultures were stimulated with media control or 10 nM ACTH at days 0, 1, 2, and 3 for the short time course and days 1, 4, 7, and 10 for the long time course. Cortisol was measured in biological duplicates using enzyme-linked immunoassay. Statistical significance: * p<0.05; † p<0.01.
