# Supplementary figures and images for: A 3D adrenocortical carcinoma tumor platform for preclinical modeling of drug response and matrix metalloproteinase activity
Source: Sci Rep. 2023 Sep 19;13:15508. doi: 10.1038/s41598-023-42659-0 (PMC10509170; doi:10.1038/s41598-023-42659-0)

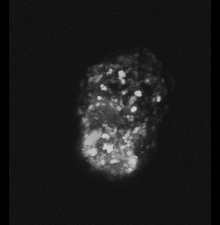

Supplement: Supplementary file 2 — Supplementary Video 1. [file 41598_2023_42659_MOESM2_ESM.gif]

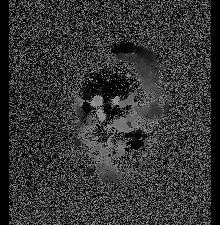

Supplement: Supplementary file 3 — Supplementary Video 2. [file 41598_2023_42659_MOESM3_ESM.gif]
